# The relationship between running distance and coaches’ perception of team performance in professional soccer player during multiple seasons

**DOI:** 10.1038/s41598-022-05519-x

**Published:** 2022-01-27

**Authors:** J. C. Ponce-Bordón, T. García-Calvo, J. M. Candela-Guardiola, F. R. Serpiello, R. López del Campo, R. Resta, J. J. Pulido

**Affiliations:** 1 Faculty of Sport Sciences, University of Extremadura, C/ Avenida de La Universidad, s/n, C.P.: 10003 Cáceres, Spain; 2 Institute for Health and Sport (IHES), Victoria University, Footscray, Australia; 3 LaLiga Sport Research Section, Madrid, Spain; 4 Faculty of Education and Psychology, University of Extremadura, Badajoz, Spain

**Keywords:** Computational biology and bioinformatics, Physiology

## Abstract

This study analyzed how the physical movement profile of soccer matches evolved throughout a season by assessing the variability of different metrics depending on the season phase. In addition, the evolution of running distances was investigated in the relation to the team performance based on the coaches’ perception. Games from four consecutives Spanish LaLiga seasons (*n* = 1520) were recorded using an optical tracking system (i.e., ChyronHego). Total distance (TD), distance covered between 14 and 21 km h^−1^ (MIRD), 21–24 km h^−1^ (HIRD), and > 24 km h^−1^ (VHIRD) were analyzed, as well as the number of efforts between 21 and 24 km h^−1^ (Sp21) and > 24 km h^−1^ (Sp24). Seasons were divided into four phases (P): P1 (matches 1–10), P2 (11–19), P3 (20–29), and P4 (30–38). Linear mixed models revealed that soccer players covered significantly greater distances and completed a higher number of sprints in P2 and P3. Also, team performance evaluated by soccer coaches was positively related to TD, HIRD, VHIRD and Sp21 in P1. A negative relationship was observed between team performance and distance covered at speeds below 21 km h^−1^ in P2 and P3. Team performance was negatively related to TD, MIRD, and HIRD, and Sp21 in P4. As conclusion, the team performance perceived by coaches is related to the movement profile throughout a season, and it significantly influences the evolution of soccer players’ movement profiles. Specifically, it seems that the players of the best teams have the best physical performance at the beginning of the season with respect to the rest of the phases.

## Introduction

In soccer, new technological advances have contributed to create new knowledge regarding the movement profiles during matches^1^. This has allowed to establish relationships between match movement profiles and different contextual-related variables^2,3^, for example final ranking or quality opponent^4^. For example, the top-ranked teams of the Spanish First Division covered significantly greater distance than the other teams belonging to First and Second Division^5^, they recorded a greater distance with the ball than the less successful teams^6^, and they show a better shooting accuracy while attacking and less shots conceded while defending^7^. Also, in the Brazilian National 2nd Division League, the top-ranked teams covered greater total distance (TD), high-speed running distance, number of sprints and high-acceleration than the bottom-ranked teams^8^. Similar results have been found establishing a positive relationship between sprinting actions and the top-ranked teams^9^. Concerning the final match status, elite international female soccer players covered greater total distance and performed higher number of sprints efforts when winning in comparison with drawing or losing^10^. During the 2018 FIFA World Cup, total distance and total distance covered sprinting were greater when the teams won^11^. Deeper analysis confirmed that winning was associated with greater total distance covered at high-intensity by wide-midfielders and forwards^12^. However there also exist conflicting results^13^, and it would be necessary to develop longitudinal analysis about team performance to analyze the evolution of team performance across seasons^4^.

For that purpose, it would be interesting to analyze whether the teams with different final performance have different match movement profiles across the season. To this extent, it is known that movement profiles vary across a season^14^. Research supports that teams who reached a better final ranking covered greater total distances^13^. Also, Lago et al.^15^ have indicated that the better the team performance at the beginning of the season, the better the ranking at the end of the season. In relation to the evolution of movement profiles within a season, it has been shown that a professional soccer team recorded the lowest TD during the preparation phase, whereas the greatest TD was achieved in mid-season (9000 vs 10,400 m)^16^. Similarly, it was found that high-intensity running distances were greater at the end of a season, with a trend from the preparation phase over the other phases of season. Similarly, Chmura et al.^14^ divided the German Bundesliga season into six phases, finding that greatest TD covered was reached in the fourth phase of the season and the lowest TD in the sixth phase (10,580 vs 10,300 m). In addition, high-intensity running distance increased over the season until the fifth phase, then decreasing in the sixth phase (240 m vs 220 m). However, these studies exclusively analyzed match movement profiles across one season, and they have not considered the relationship between match movement profiles and final team performance.

Based on the gaps of the aforementioned studies, we aimed to analyze (i) how the match movement profiles evolve within season (ii) and across seasons (into four different phases), and (iii) how running distance variables could be related to the coaches’ perceptions of team performance. To this extent, the use of final ranking to quantify team performance may not seem a good indicator due to the multi-faceted nature of soccer and the initial different goals of teams according their budget and characteristics^4,17^. For that reason, previous literature has used an alternative way of measuring the teams’ success such as expected goals model^18^. In this study, we have used another teams’ evaluation approach like coaches’ perceptions of team performance, which have been previously considered useful^19^. Specifically, coaches were asked to assess the team performance considering the final ranking of each team at the end of each season. Additionally, they were informed about the budget and the characteristics regarding the players of each team. Considering these three main aspects, they rated the performance of each team in each season. Therefore, considering four consecutive seasons of Spanish LaLiga, this study aimed to analyze whether the relationship between team performance based on the expert coaches´ team performance assessment and the evolution of movement profiles in different phases across season was similar among teams. Based on prior findings obtained by previous studies, the following hypotheses were proposed: (i) we considered that a variability between teams exists^20^; (ii) concerning the evolution on movement profiles, we expected that total distance, the distance covered at high intensity, and the number of very high-intensity running efforts would be higher in middle-season (Phase 2, and Phase 3)^14,16^; (iii) we expected that the best-teams recorded greater total distance and higher high-intensity running distance at the early-season than the bottom-ranked teams, since previous studies has shown that successful teams at early-season reached first positions in the final ranking^15^.

## Methods

### Participants and procedure

The sample was composed of 1520 matches played by 80 professional soccer teams across four consecutive seasons of LaLiga (from 2015/2016 to 2018/2019). Two observations were collected per match, one from each team, resulting in a total of 2950 records (760 per season). A total of 90 (11.82%) recordings were excluded (2015/16 = 10 (1.31%); 2016/17 = 16 (2.10%); 2017/2018 = 35 (4.60%); 2018/19 = 29 (3.81%)) due to issues related to repeated signal loss by the system or adverse weather conditions during the match that hindered accurate data collection. Data were provided to the authors by LaLiga™ after four consecutive seasons were concluded (See supplementary file). All players were also informed about the study’s protocol. Written informed consent was obtained from all the participants and from a parent and/or legal guardian for subjects under 18. The study received ethical approval from the University of Extremadura; Vice-Rectorate of Research, Transfer and Innovation—Delegation of the Bioethics and Biosafety Commission (Protocol number: 239/2019).

### Variables

#### Match movement profiles

The distance covered by teams were analyzed with Mediacoach^21,22^ in different speed ranges: total distance covered by teams in meters (i.e., TD); distance covered by teams between 14 and 21 km h^−1^ (i.e., MIRD = Medium-intensity running distance); between 21 and 24 km h^−1^ (i.e., HIRD = High-intensity running distance); and above 24 km h^−1^ (i.e., VHIRD = Very high-intensity running distance), as well as the number of high-intensity efforts performed: number of very high-intensity running efforts between 21 and 24 km h^−1^ (i.e., Sp21); and number of sprints > 24 km h^−1^ (i.e., Sp24). Mediacoach is an analysis software utilising the tracking data provided from a third-party tracking system (ChyronHego), which consists of a series of super 4 K-High Dynamic Range cameras that film from several angles and analyze X and Y coordinates of each player. This instrument is also based on the correction of the semi-automatic video tracking system (the manual part of the process). The validity and reliability of the ChyronHego system have been previously tested by other studies^21,23–26^, reporting average measurement errors of 2% for total distance covered.

#### Season phases

Similar to previous research^14^, every season was split into four blocks of matches to allow for easier interpretation of the data. Specifically, according to the number of matches per season of Spanish LaLiga, every season was split into four phases: matches 1–10 (i.e., Phase 1 = P1), matches 11–19 (i.e., Phase 2 = P2), matches 20–29 (i.e., Phase 3 = P3), and matches 30–38 (i.e., Phase 4 = P4).

#### Team performance perceived by experts’ soccer coaches

The performance of each team was evaluated by twenty soccer coaches with a minimum of five years of experience since obtaining their UEFA PRO qualification. Additionally, all coaches had coached professional teams for at least one full season and had no contractual relationship at the time of data collection with any of the teams which were included the current study. Once each season concluded, coaches individually evaluated the teams’ performance considering three main elements: final ranking achieved by team in each season, team characteristics (e.g., quality of the players), and the total budget available provided by LaLiga. To this end, a protocol was followed to create the questionnaire used to gather the coaches’ opinion. First, a panel of experts composed by three sport researchers individually proposed three aspects to assess the team performance. A think-aloud protocol of the three proposals was held to highlight the different points from the experts’ perspective. Next, doubts and discrepancies between experts were discussed to arrive at consensual agreement on the final proposal^27^. Finally, the final version was reviewed and unanimously accepted, and coaches provided an individual score for each team and each season considering the three elements and using a 10-point Likert-type scale from 1 (*very low performance*) to 10 (*very high performance*). The ratings were then categorized into low performance (1–4), medium performance (5–7), and high performance (8–10). The intraclass correlation coefficient (*ICC*)—calculated to test inter-rater reliability was 0.92.

### Statistical analysis

Data were analyzed using R Studio^28^. (i) Considering the hierarchical data structure (as matches were nested into teams), six unconditional models were analyzed (see Table 1) exclusively including dependent variables (i.e., match movement profiles variables). ICC showed values greater than 10% in all variables. (ii) Due to this reason, six linear mixed models were calculated (see Table 2) to compare the match movement profiles variables in the four phases considered (i.e., P1, P2, P3, and P4), including the intercept of the teams as random effects^29^.

**Table 1:** Unconditional model results of match movement profiles for Spanish soccer teams.

Variables	TD (m)	MIRD 14–21 km h^−1^ (m)	HIRD 21–24 km h^−1^ (m)	VHIRD > 24 km h^−1^ (m)	No. Sp 21–24 km h^−1^	No. Sp > 24 km h^−1^
	Coeff	Coeff	Coeff	Coeff	Coeff	Coeff
**Fixed effects**						
Intercept	109,117***	22,428***	3018***	2904.66***	264.83***	161.02***
**Random effects**						
Residual	13,716,148***	3,143,788***	125,973***	200,424***	786.21***	427.77***
Intercept	5,285,581***	1,627,025***	23,308***	40,998***	166.70***	104.06***
*ICC*	0.28	0.34	0.16	0.17	0.17	0.20
AIC	57,021.31	52,701.91	43,132.01	44,506.99	28,185.80	26,402.45

*m* meters, *No.* number, *TD* total distance, *MIRD* medium-intensity running distance, *HIRD* high-intensity running distance, *VHIRD* very high-intensity running distance, *Sp21-24* Sprints between 21 and 24 km h^−1^, *Sp > 24* Sprints at speeds above 24 km h^−1^, *Coeff* coefficient, *ICC* Intraclass Correlation Coefficient, *AIC* Akaike Information Criteria.

*p < 0.05, **p < 0.01, ***p < 0.001.

**Table 2:** Linear mixed model results of match movement profiles by season phases for Spanish soccer teams.

Variables	TD (m)		MIRD 14–21 km h^−1^ (m)		HIRD 21–24 km h^−1^ (m)		VHIRD > 24 km h^−1^ (m)		No. Sp 21–24 km h^−1^		No. Sp > 24 km h^−1^	
	Coeff	*p*	Coeff	*p*	Coeff	*p*	Coeff	*p*	Coeff	*p*	Coeff	*p*
**Fixed effects**												
Intercept Phase 1	108,423	bc***d*	21,640	bcd***	2887	bcd***	2775	bcd***	254.32	bcd***	154.63	bcd***
Phase 2 – Phase 1	1225	a***d**	1256	ad***	218	ad***	184	a***	17.69	ad***	10.12	a***d**
Phase 3–Phase 1	1049	a***d*	1253	ad***	203	ad***	194	a***d*	16.70	ad***	9.74	a***d**
Phase 4–Phase 1	591	a*b**c*	735	abc***	120	abc***	149	a***c*	8.97	abc***	6.48	a***b**c**
**Random effects**												
Residual variance	12,567,375	***	2,637,957	***	113,957	***	189,037	**	710.13	***	402.17	***
Intercept	5,014,165	***	1,569,324	***	22,322	***	39,744	**	160.91	***	102.19	***
Phases	1,210,464	***	299,740	***	5492	**	6697	**	31.32	**	11.23	*
AIC	56,883.53		52,335.87		428,908.71		443,381.72		28,052.62		26,351.19	
Chi Square	48.92		265.01		186.41		94.31		199.94		118.66	

**Figure 1:**
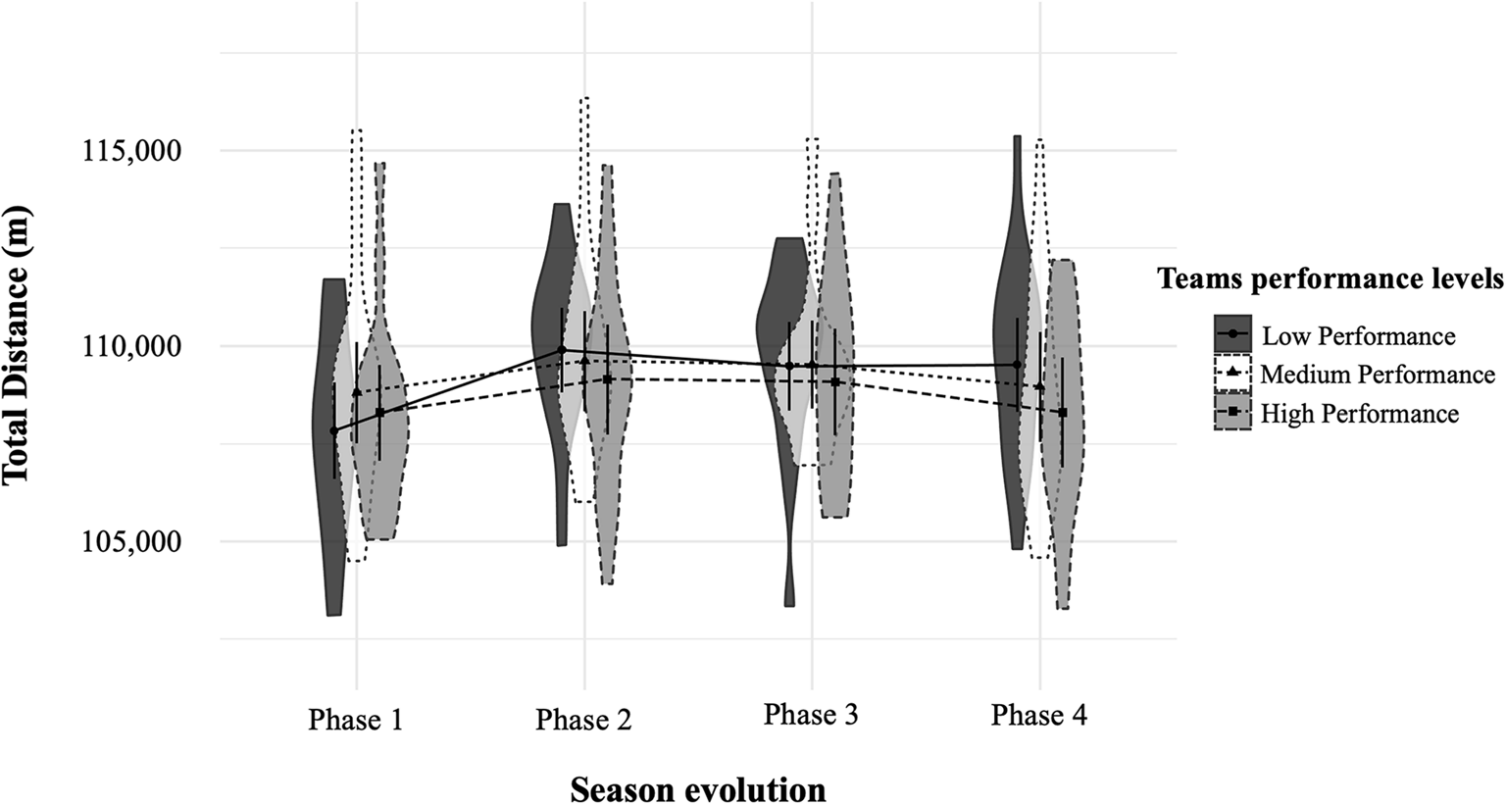
Total distance covered in meters (*M* ± *SD*) by teams with high, medium and low performance in the different phases of the seasons^29^. *m* meters.

**Figure 2:**
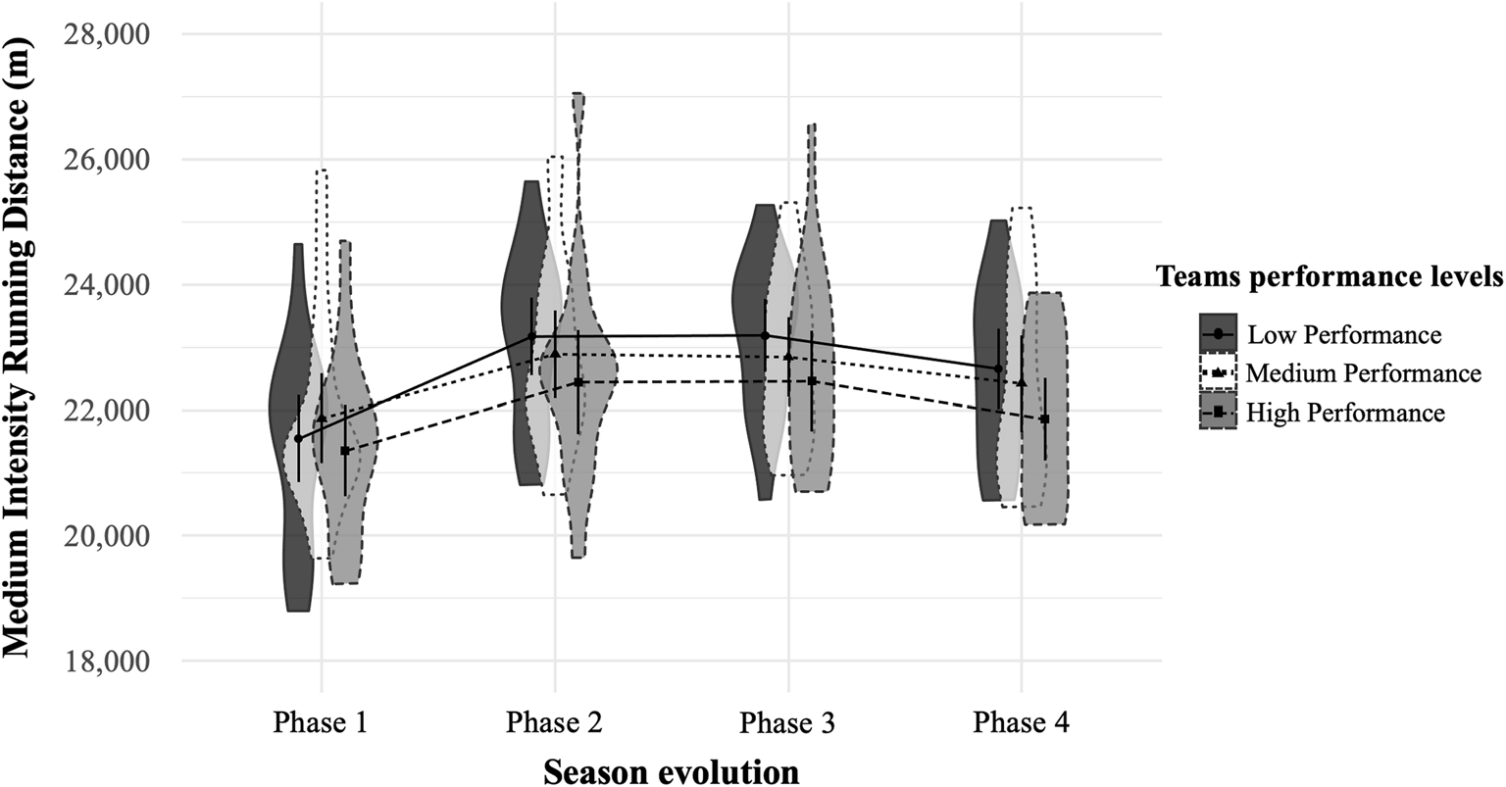
Medium intensity running distance (14–21 km h^−1^) covered in meters (*M* ± *SD*) by teams with high, medium and low performance in the different phases of the seasons^29^. *m* meters.

**Figure 3:**
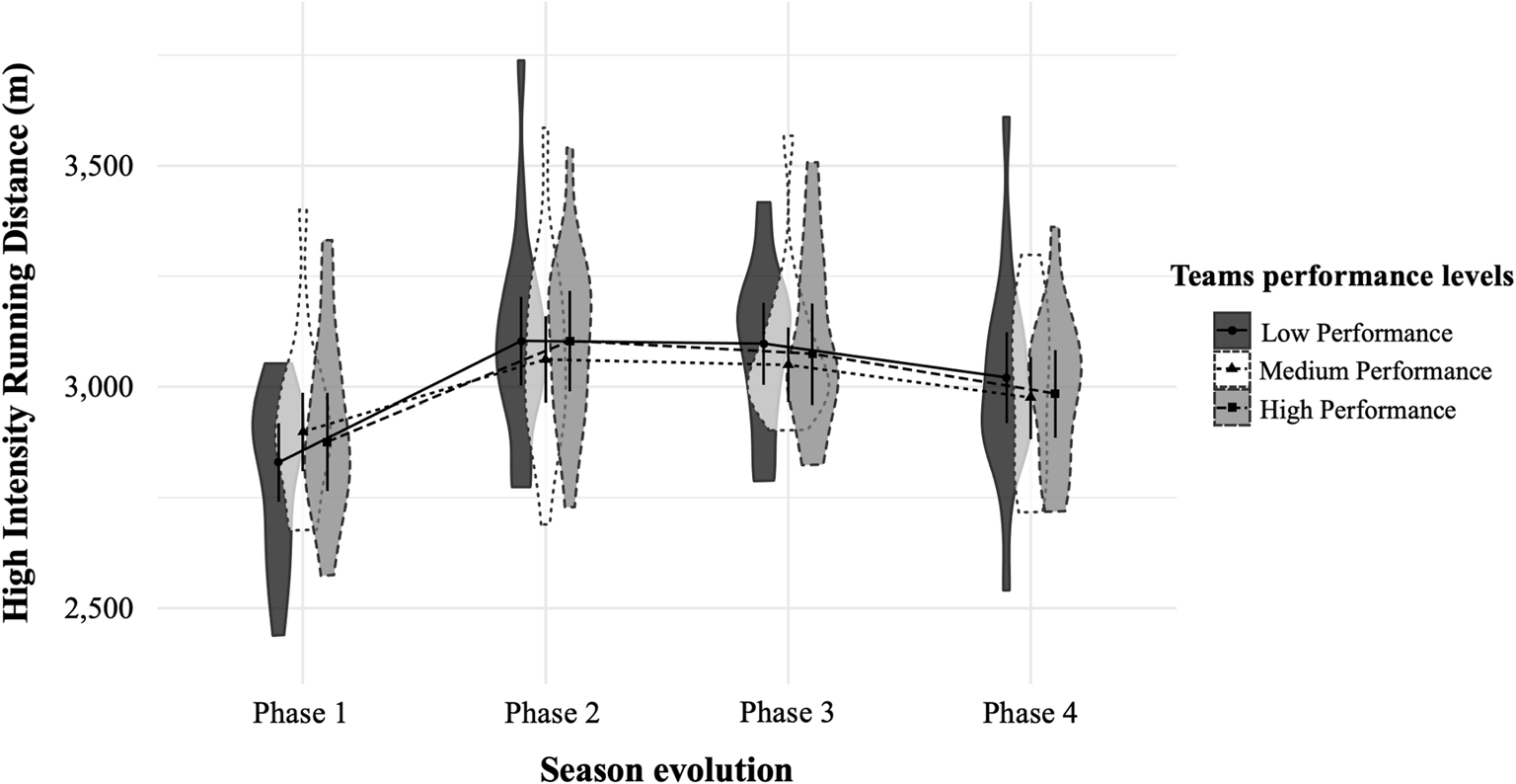
High intensity running distance (21–24 km h^−1^) covered in meters (*M* ± *SD*) by teams with high, medium and low performance in the different phases of the seasons^29^. *m* meters.

**Figure 4:**
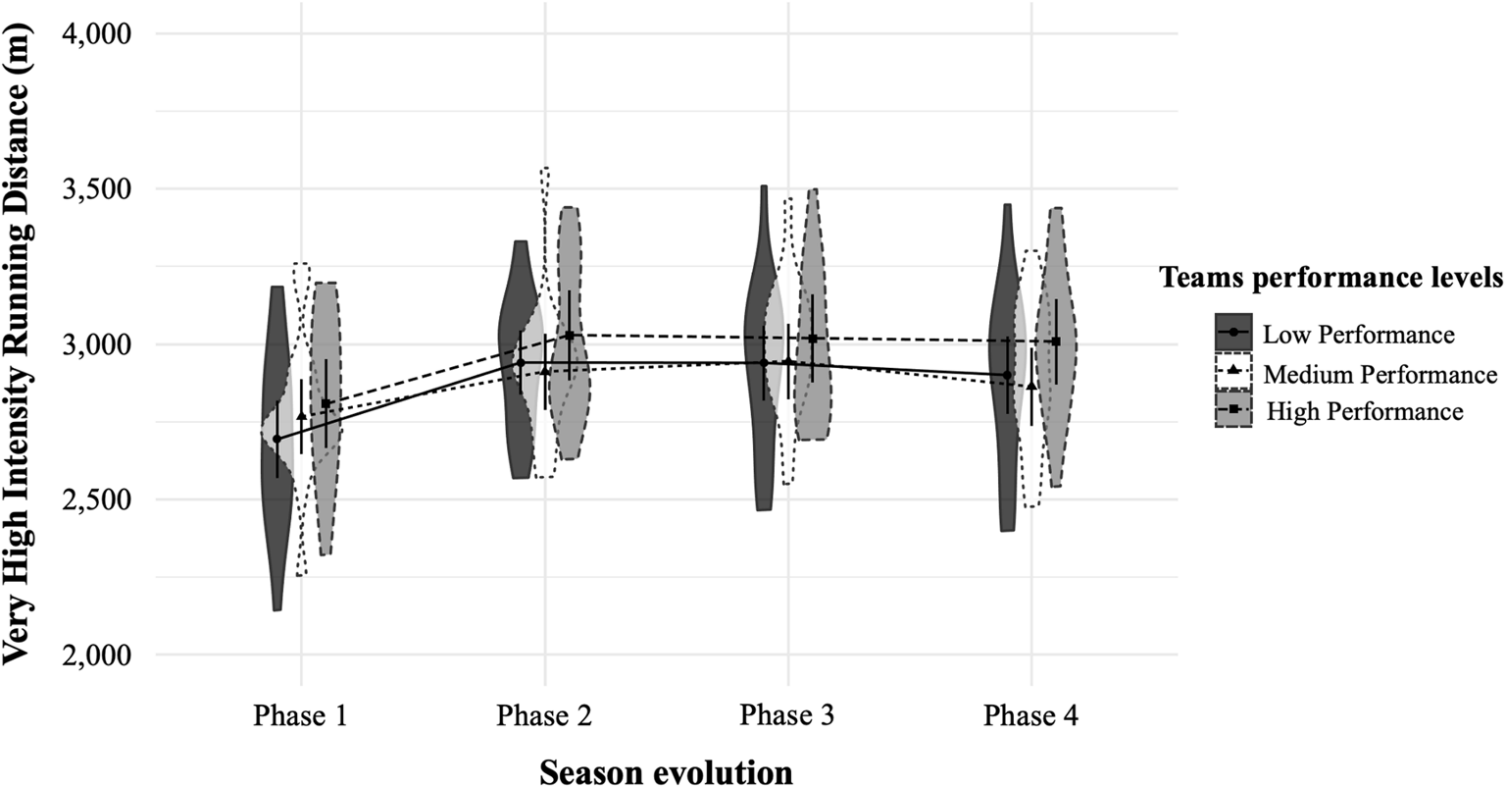
Very high-intensity running distance (> 24 km h^−1^) covered in meters (*M* ± *SD*) by teams with high, medium and low performance in the different phases of the seasons^29^. *m* meters.

**Figure 5:**
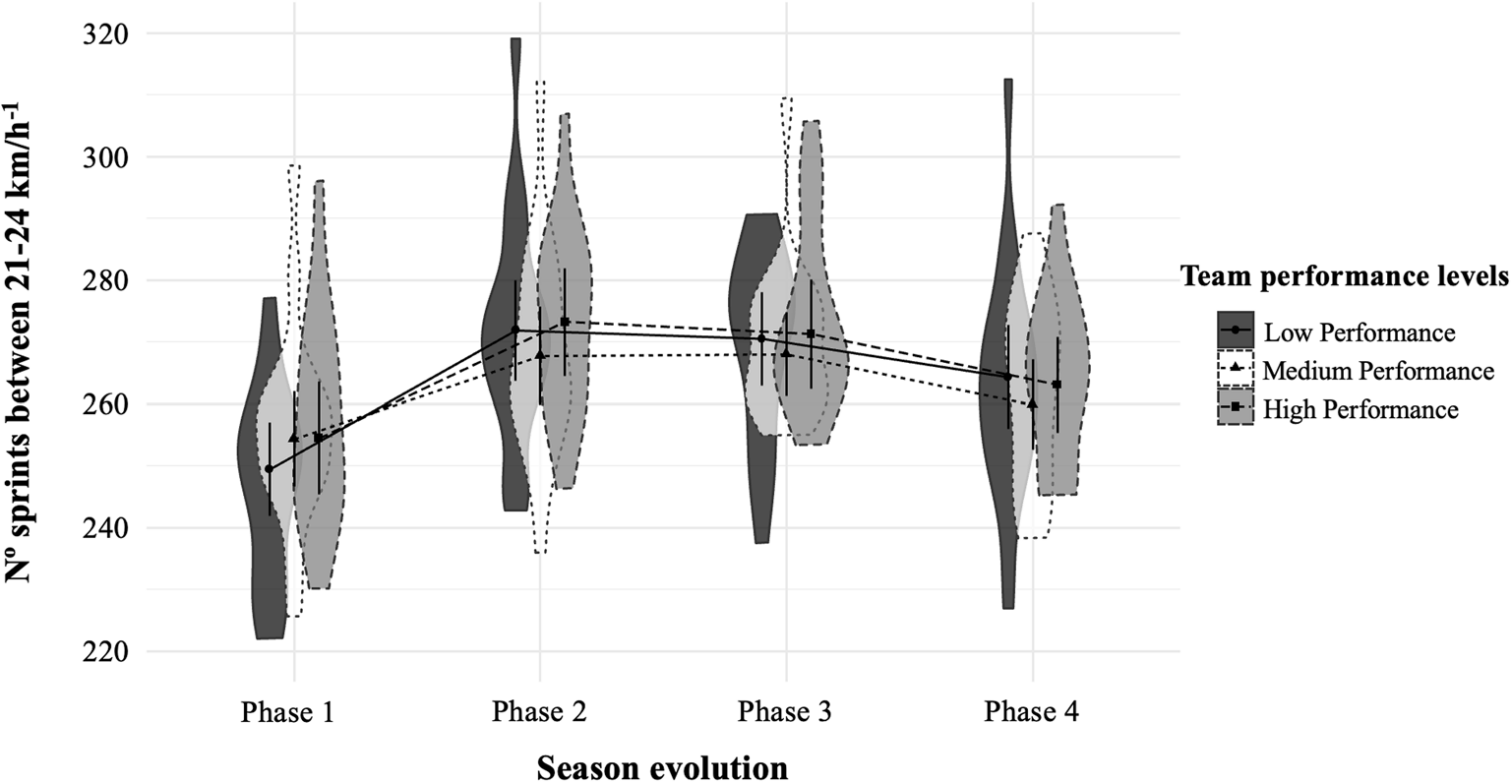
Number of sprints between 21 and 24 km h^−1^ registered (*M* ± *SD*) by teams with high, medium and low performance in the different phases of the seasons^29^.

**Figure 6:**
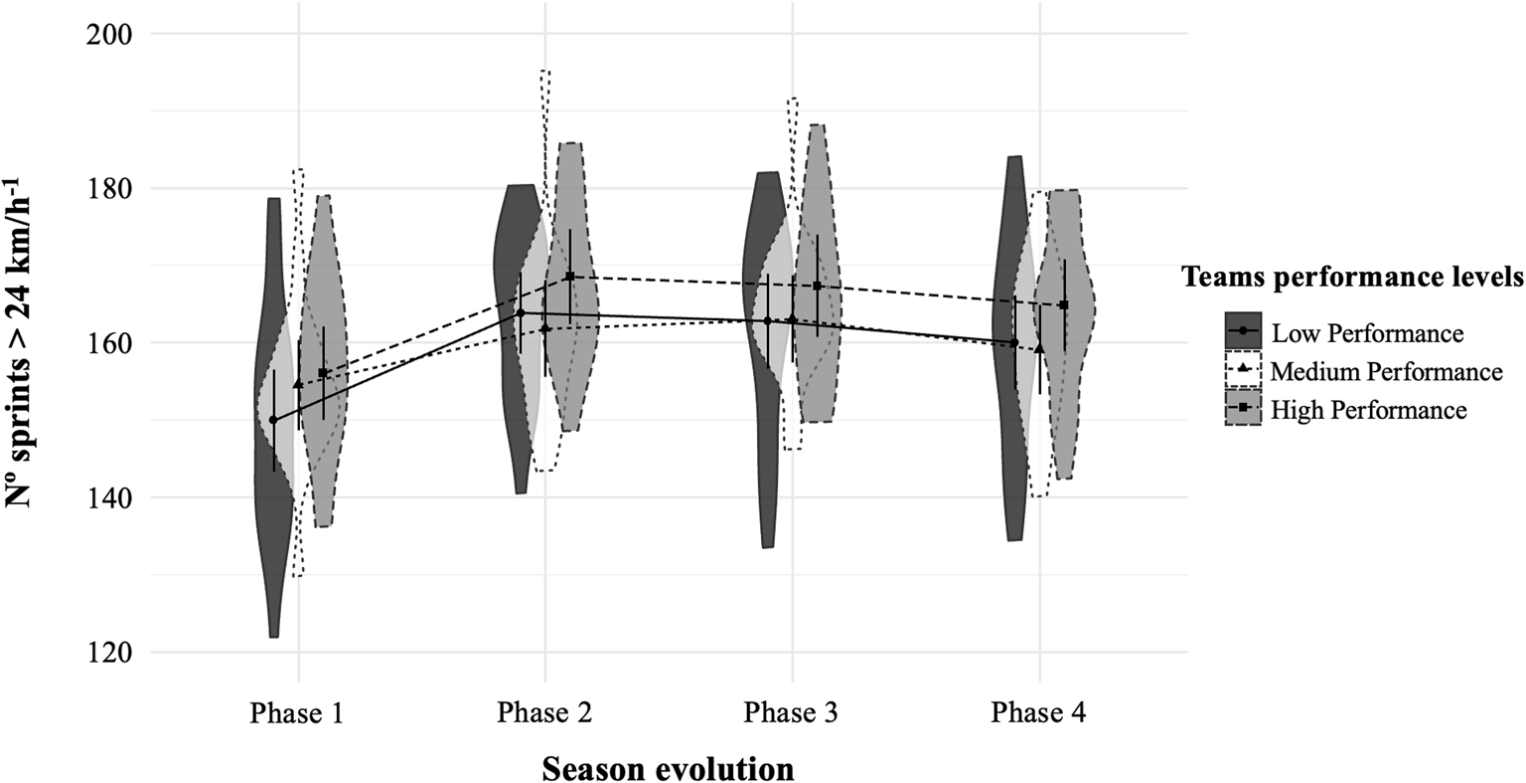
Number of sprints > 24 km h^−1^ registered (*M* ± *SD*) by teams with high, medium and low performance in the different phases of the seasons^29^.

*p < 0.05, **p < 0.01, ***p < 0.001—comparing the six models in the different phases of the season, *m* meters, *No.* number, *TD* total distance, *MIRD* medium-intensity running distance, *HIRD* high-intensity running distance, *VHIRD* very high-intensity running distance, *Sp21-24* Sprints between 21 and 24 km h^−1^, Sp > 24 = Sprints at speeds above 24 km h^−1^, *Coeff.* Coefficient, a = differences with Phase 1; b = differences with Phase 2; c = differences with Phase 3; d = differences with Phase 4.

(iii) Another linear mixed model was estimated for each dependent variable (see Table 3), with random intercepts and slopes. First, the different season phases (i.e., P1, P2, P3, and P4) were included as factor and team performance perceived by soccer coaches as covariate of match movement profiles variables^30^. Finally, to facilitate a clearer interpretation of the results presented in Table 3 and through linear mixed model analysis, six figures (one for each dependent variable; see Figs. 1, 2, 3, 4, 5, 6) were represented. Specifically, we included the interaction between team performance perceived by experts (i.e., high, medium, and low team performance levels) and the different phases of the season (i.e., P1, P2, P3, and P4) on the different movement profiles ranges. These figures were performed using the 0.14.1 version of software JASP (https://jasp-stats.org/)^31^.

**Table 3:** Linear mixed model results of match movement profiles by team performance and season phases for Spanish soccer teams.

Variables	TD (m)	MIRD 14–21 km h^−1^ (m)	HIRD 21–24 km h^−1^ (m)	VHIRD > 24 km h^−1^ (m)	No. Sp 21–24 km h^−1^	No. Sp > 24 km h^−1^
	Coeff	Coeff	Coeff	Coeff	Coeff	Coeff
**Fixed effects**						
Intercept						
Phase 1	108,424***	21,640***	2887***	2776***	254.35***	154.64***
Phase 2–Phase 1	1223***	1255***	218***	184***	17.67***	10.11***
Phase 3–Phase 1	1046**	1252***	203***	194***	16.69***	9.73***
Phase 4–Phase 1	588*	734***	120***	149***	8.95***	6.48***
Slope						
Soccer coaches´ perception of team performance*Phase 1	108^†^	20	22*	22^†^	2.21*	1.22
Soccer coaches’ perception of team performance*Phase 2	− 296*	− 117*	− 13	− 12	− 1.43	− 0.77
Soccer coaches´ perception of team performance*Phase 3	− 240*	− 112^†^	− 15	− 12	− 1.32	− 0.64
Soccer coaches´ perception of team performance*Phase 4	− 428***	− 155**	− 25*	− 14	− 2.09**	− 0.84
**Random effects**						
Residual variance	12,568,737***	2,638,145***	113,970.92***	189,046***	710.23***	402.29***
Intercept	5,039,002***	1,570,447***	22,405.87***	39,587***	159.64***	101.82***
Slope	1,094,986***	287,206***	5223.16**	6845**	29.15**	11.24*
AIC	56,823.68	52,287.20	42,876.37	44,352.00	27,952.92	26,268.11
Chi Square	73.93	281.38	198.24	98.65	212.84	123.89

*m* meters, *No.* number, *TD* total distance, *MIRD* medium-intensity running distance, *HIRD* high-intensity running distance, *VHIRD* very high-intensity running distance, *Sp21-24* Sprints between 21 and 24 km h^−1^, Sp > 24 = Sprints at speeds above 24 km h^−1^, *Coeff.* Coefficient.

^†^*p* < 0.07, *p < 0.05, **p < 0.01, ***p < 0.001.

The Akaike Information Criteria (AIC) and Chi square were considered to compare the unconditional models with linear mixed models fit. AIC is an estimate of the mean log likelihood and provides a versatile procedure for statistical model identification. The model goodness-of-fit is higher as the statistical value decreases^32^. Chi square provides information on the magnitude of the differences between the models. In all the variables analyzed, the best models were those that included perceived performance and the season phases.

### Ethics approval

The procedure was carried out according to the guidelines and regulations provided by the institutional ethical approval (protocol number 239/2019).

## Results

### Main analysis

(i) Six unconditional models including the distances covered in the different speed ranges and the number of Sp2 and Sp24 were obtained (Table 1). Intercepts represent the estimated mean of each variable. For both fixed and random effects, significant variability was detected for all dependent variables (*p* < 0.001).

(ii) Table 2 contains the linear mixed models including the distance covered and the number of sprints in different speed ranges considering the four phases in which each season was split (i.e., P1, P2, P3, and P4). Significant differences were found in all dependent variables when comparing P1 to the rest of phases (*p* < 0.05). In particular, shorter distances in meters and a lower number of sprints in the different speed ranges were obtained in the first part of the season. Considering P2 and P3 as references, significant differences were also found compared to P4 in TD, MIRD, HIRD, VHIRD (not comparing P2 to P4) and in the number of Sp21 and Sp24 (*p* < 0.05). In other words, soccer players covered significantly greater distances and performed a higher number of sprints in P2 and P3 compared to P1 and P4, respectively.

Regarding random effects, significant differences were observed in the distance covered by teams (*p* < 0.001), as well as in the evolution (i.e., slopes) throughout the season phases (*p* < 0.001).

(iii) Table 3 shows the linear mixed models predicting the distance covered and the number of sprints in the different speed ranges considering the season phases (i.e., P1, P2, P3, and P4) and team performance perceived by soccer coaches. Team performance was positively related to the distance covered in all speed ranges (e.g., TD increased by 108.34 m when performance increased by 1 point) and to the number of Sp21 and Sp24 in P1 (*p* < 0.001). That is, the better the team performance, the longer the distance covered and the higher the number of sprints completed at the beginning of the season. Nevertheless, this was only significant for HIRD (*p* < 0.05) and the number of Sp21 (*p* < 0.05). In P2 and P3, a negative relationship was detected between team performance on one hand and the distance covered in the different speed ranges and the number of sprints on the other. However, significant decreases compared to P1 (*p* < 0.05) were only found in distance covered at speeds below 21 km h^−1^ (e.g., TD = − 300.41 m and − 240.49 m, respectively). Finally, team performance perceived by experts was also negatively related to the distance covered in all speed ranges and the number of Sp21-24 (8.95; *p* < 0.001) and Sp > 24 (6.48; *p* < 0.001) in P4. However, significant negative relationships were only found in TD (*p* < 0.001), MIRD (*p* < 0.01), and HIRD (*p* < 0.05), as well as in the number of Sp21 (*p* < 0.001). Thus, the better the team performance during the last season phase, the shorter the distance covered at low and medium intensities (e.g., high team performance = − 436.72 m in TD in P4 compared to P1) and the lower the number of sprints performed at speeds between 21 and 24 km h^−1^, compared to P1 (*p* < 0.05).

As regards random effects, residual variances (e.g., Sp21 = 710.23, *p* < 0.001) and intercepts (e.g., Sp21 = 159.64, *p* < 0.001) were significant for all dependent variables (*p* < 0.001). The evolution of these associations from phase to phase of the season was also estimated. The slopes of all distances covered and number of sprints changed significantly (*p* < 0.05). AIC values for all dependent variables were lower than AIC of unconditional models and the six models before, respectively. Likewise, the values obtained in the chi-square deviance testing report that this is the model with the best fit with respect to the previous models.

Finally, Figs. 1, 2, 3, 4, 5, 6 illustrate the results given in Table 3 with respect to the relationship between team performance perceived by soccer coaches (i.e., high, medium, and low performance) and the distance covered in the different speed ranges in the different season phases (i.e., P1, P2, P3, and P4). Specifically, teams with high performance recorded higher VHIRD (2800 m) and number of Sp24 (156) at early-season, and less TD (108,303 m) and MIRD (22,004 m) at the end-season than the rest of teams. However, teams with low performance recorded less TD (108,012 m), VHIRD (2738 m), Sp21 (249), and Sp24 (152) at early-season, and higher TD (109,958 m), MIRD (22,688 m), and HIRD (3021 m) at end-season than the rest of teams.

## Discussion

The present study aimed to analyze the variability among the teams’ movements profiles and whether the team performance and the evolution of movement profiles in the different phases could explain this variability. The relationship between the evolution of movement profiles and team performance was examined by verifying that team performance could explain the differences in match movement profiles using a multilevel perspective.

The main findings of the study were: (i) running distances covered at different intensities varied significantly throughout each season; (ii) running distances covered were significantly higher in the mid-season (i.e., P2 and P3) at all intensities. Despite these results have been already checked by previous studies^14,16^, this study intended to be a first insight in how team performance perceived by coaches could be another variable that explain why soccer players run differently across season. Accordingly, (iii) team performance based on the judgement of expert coaches was positively and significantly related to the distances covered by teams in the early phase of the season, however, this relationship was negative and significant in P2, P3, and P4. In other words, having a better team performance was associated with greater distances in the early-season and covered less distance at the end-season (or vice versa).

Regarding the first contribution of this study, the results revealed variability within teams and among teams throughout the season. In particular, random effects indicate that there are differences between and within teams in the evolution throughout the different season phases. On one hand, the variability in team physical performance may be associated with the different contextual-related variables^2^, or with the tactical changes that occur during a match^11^. It may also be related to technical-tactical variables, such as team ball possession^33,34^. Also, it could be related to the team performance^4^. Likewise, Table 2 shows the variability in the different season phases, so the match movement profiles of soccer players are different throughout the four phases.

Secondly, as we expected, TD covered by teams increased progressively during the middle-season and then it slightly decreased, revealing that teams covered the shortest distances at the early-season. In general, it can be stated that soccer players covered shorter distances in matches of early-season, and these distances progressively increase over the middle-season and decrease again at the end-season. A possible explanation may be due to a decrease in physical performance during the detraining period (i.e., off-season), as previously revealed other studies, since some weeks are necessary to improve fitness levels of soccer players^35,36^. Furthermore, during preseason phase is employed a workload meaningfully greater than in the rest of season^37^, causing a state of physical and mental fatigue, what could be the potential cause of a lower physical performance at early-season^38^. During the middle-season phases (i.e., P2 and P3), the distances covered by teams significantly increased. These results are in line with studies that showed that total distance covered by teams significantly increased during middle-season^14,16,39^. Adaptation to training load and to competition demands as the season progresses could be key factors to understand the increase in physical performance during the middle-season^40^.

By contrast, the distances covered in the different speed sections and the number of sprints decreased during P4 compared to middle-season phases (i.e., P2 and P3). On contrary by previous studies^39^, where only one team was analyzed and the season phases were longer, our results suggested a decrease in this type of actions at end-season, although some teams kept these values during whole season. There are several potential causes that could explain this decrease. Previous studies have reported that accumulated fatigue throughout competition and subsequent incomplete recovery could produce a decrease in physical performance towards the end-season^35,41^, due to the difficulty to maintain physical fitness levels during long periods of time^42^. Another possible cause of this may be due to the improvement players’ tactical performance achieved during the season affect physical demands, so that their tactical movements become more efficient and distances covered decrease^43,44^. The decrease of high-intensity running actions could be a consequence of a reduction in match intensity, since some teams have already accomplished their goals and it exists a lack of motivation, what may affect the distances covered and the number of sprints performed during the end-season^45^.

Thirdly, considering the variability observed during the season and as we hypothesized, coaches’ perception of the team performance was positively related to the distances covered by teams in the different speed ranges in early-season (i.e., Phase 1). However, as the season progressed, this relationship turned to negative in the most variables. In this regard, it can be stated that better team performance perceived by experts was related to greater movement profiles in early-season at all the intensities analyzed, the relationship being stronger at high intensities. Conversely, as the season progressed, the relationship between the team performance and movement profiles inverted. Consequently, the distances covered and the number of sprints performed significantly decreased as team performance increased in P4 compared to the early-season (i.e., Phase 1). That is, the better the team performance based on experts’ perceptions, the shorter they ran in the last ten matches of the season. That is, the worse the team performance perceived by coaches, they covered greater distances in the end-season at all intensities analyzed, except number of Sp21and Sp24. The positive relationship between successful teams and greater distances covered and a higher number of sprints performed at the early-season could be explained due to their players presented better physical fitness levels that allowed them to carry out a better adaptation to training and competition demands. In addition, these teams were able to reach such physical performance level because they got fitter better than the rest due to the training program they followed during the pre-season. This enabled them to travel greater distances and complete a higher number of sprints than the rest of teams^37^. It has been proved that the outcome of the first matches have relevant influence on the final ranking^15,46^. So, starting the season being fit could lead these teams to earned greater average point at the early-season. According to our results, the most successful teams recorded the longest distances and performed the highest number of sprints during the early-season. Our findings agree with those from other studies where positive relationship was observed between team final ranking and the sprinting activity covered during the season^9^. However, these studies measured the team performance exclusively based on the final ranking.

By contrast, the least successful teams by the end-season were those who recorded the shortest distances at all intensities in P1 compared to the rest of phases (P2, P3, and P4). During the end-season phase (P4), a negative relationship was observed between distances covered and team performance perceived by soccer coaches. This means that less successful teams covered greater distances than the rest of teams, reaching higher values than in the rest of phases (P1, P2, and P3). This could be explained due to the need that arises at the end-season to accomplish the goals established at the early-season. Thus, less successful teams try by all means to fulfill these goals, what produces greater competitive stress, leading to greater distances covered. Previously it has been demonstrated that when a team approached bottom-ranked, they covered greater distances than when they were in a more comfortable position^47^. According to the present study, during the end-season phase, less successful teams presented higher TD, MIRD, and HIRD, as well as a higher number of Sp21. Nonetheless, although team performance has not been assessed through final ranking in our study, teams who were struggling to avoid relegation had probably not achieved the expected performance level. On the other hand, the increase in the distances covered by soccer players may indicate the effect of less synchronization after a congested period during the middle and end-season and their team performance may decrease. Thus, those teams who are not able to maintain their tactical synchronization could cover greater distances and may show worse performance^44,48^. One potential reason for this tactical variability and, in turn, the greater distances covered, may be coach turnover, in an effort to revert the negative situation^49^.

## Conclusion

The present research represents an attempt to determine how running performance evolves in professional soccer teams. Firstly, there is variability between distances covered by teams, meaning, the distances covered by teams were different and this variability affected the evolution of match movement profiles. Specifically, the different distance variables changed throughout the season, the shortest distances at all intensities having been traveled at the early and end-season. Despite there is variability between teams in the different phases of the season, the team performance is one factors that explains this variability. There was a positive relationship between the distances covered and team performance at the early-season. Therefore, teams with best team performance reached at end-season covered higher distances at early-season. Thus, the performance level achieved at the end-season may explain the covered distance variability among teams throughout evolution of different season phases.

## Limitations and future directions

A number of limitations could be recognized after developing the current study. First, situational variables such as match status, match outcome, match location or opposing team’s level, or even technical-tactical variables were not analyzed. Coach turnover, which may influence to the team tactical and physical performance^49^, was not examined either. Second, other official competitions played by some teams were not considered. Data may have been affected by accumulated fatigue in teams who played other competitions (European competitions or National cup—knockout competition—) that were not controlled. Nevertheless, a previous study revealed that a congested competition calendar did not affect to the team physical performance^50^. Finally, results of the present study might be biased due to the fact that coaches’ impressions might have been affected by the team performances in the first part of the season. Therefore, in further studies it would be interesting to carry out a bidirectional relationship (i.e., across season and after season) to avoid the early impressions.

## Practical applications

The present study could help to both the head coaches and the rest of coaching members to adjust the training load to be applied during the pre-season and season. Also, the practitioners could consider that the loss of fitness level presented at the early-season should be minimized in order to start the season in optimal conditions^51^, although the physical levels may also decrease due to excessive or inappropriate training load in the pre-season. Another practical implication of the current study could be the way in which the team performance was measured based on the coaches’ judgement considering the final ranking and the budget of the clubs. This represents an alternative way (different from traditional assessment, like as final ranking) of assessing team performance that allow for further studies. Finally, considering that the distances covered through the season are different among teams and within teams in the four parts in which the seasons were divided, the head coaches and the rest of the coaching staff could use the specific information of their teams in order to program training load in a more strategic way based on data. In this vein, technical staff could plan harder or softer training sessions regarding total distance covered, distance covered at high intensity or number of high-intensity efforts considering the season phase. Because unsuitable training loads could cause inability players to correctly perform season phase.

## Supplementary Information

Supplementary Information.

## Data Availability

Restrictions apply to the availability of these data. Data was obtained from LaLiga and are available at https://www.laliga.es/en with the permission of LaLiga.
